# Theoretical Perspectives Underpinning Research on the Physician-Patient Relationship in a Digital Health Practice: Scoping Review

**DOI:** 10.2196/47280

**Published:** 2024-05-15

**Authors:** Damoun Nassehi, Birgitta Haga Gripsrud, Ellen Ramvi

**Affiliations:** 1 Department of Caring and Ethics Faculty of Health Sciences University of Stavanger Stavanger Norway

**Keywords:** digital health, mobile health, telemedicine, physician-patient relations, relational ethics, primary care, patient-provider, physician-patient, telehealth, relationship, eHealth, scoping review, review method, mobile phone

## Abstract

**Background:**

The advent of digital health technologies has transformed the landscape of health care, influencing the dynamics of the physician-patient relationship. Although these technologies offer potential benefits, they also introduce challenges and complexities that require ethical consideration.

**Objective:**

This scoping review aims to investigate the effects of digital health technologies, such as digital messaging, telemedicine, and electronic health records, on the physician-patient relationship. To understand the complex consequences of these tools within health care, it contrasts the findings of studies that use various theoretical frameworks and concepts with studies grounded in relational ethics.

**Methods:**

Using the PRISMA-ScR (Preferred Reporting Items for Systematic Reviews and Meta-Analyses extension for Scoping Reviews) guidelines, we conducted a scoping review. Data were retrieved through keyword searches on MEDLINE/PubMed, Embase, IEEE Xplore, and Cochrane. We screened 427 original peer-reviewed research papers published in English-language journals between 2010 and 2021. A total of 73 papers were assessed for eligibility, and 10 of these were included in the review. The data were summarized through a narrative synthesis of the findings.

**Results:**

Digital health technologies enhance communication, improve health care delivery efficiency, and empower patients, leading to shifts in power dynamics in the physician-patient relationship. They also potentially reinforce inequities in health care access due to variations in technology literacy among patients and lead to decreases in patient satisfaction due to the impersonal nature of digital interactions. Studies applying a relational ethics framework have revealed the nuanced impacts of digital health technologies on the physician-patient relationship, highlighting shifts toward more collaborative and reciprocal care. These studies have also explored transitions from traditional hierarchical relationships to mutual engagement, capturing the complexities of power dynamics and vulnerabilities. Other theoretical frameworks, such as patient-centered care, and concepts, such as patient empowerment, were also valuable for understanding these interactions in the context of digital health.

**Conclusions:**

The shift from hierarchical to collaborative models in the physician-patient relationship not only underscores the empowering potential of digital tools but also presents new challenges and reinforces existing ones. Along with applications for various theoretical frameworks and concepts, this review highlights the unique comprehensiveness of a relational ethics perspective, which could provide a more nuanced understanding of trust, empathy, and power dynamics in the context of digital health. The adoption of relational ethics in empirical research may offer richer insights into the real-life complexities of the physician-patient relationship, as mediated by digital technologies.

## Introduction

### Rationale

Digital health technologies are revolutionizing the practice of health care [1]. Driven by societal, political, and technological advancements, these technology-mediated changes are occurring at an unprecedented pace. Envisioning a future in which medicine can be personalized to individuals and diseases can be foreseen and prevented, some experts have high hopes for the potential of technology to improve human life [1,2]. However, skeptics argue that achieving personalization through technology is an elusive paradox, as it fails to account for the fundamental uncertainties and complexities of medicine and the value of human interactions [3].

In the realm of medical practice, a physician’s primary goal is to diagnose and treat patients’ diseases by drawing upon their biomedical expertise and years of clinical experience. Nevertheless, a physician’s practice must also be guided and adapted based on their relationship with the patient. A sustained physician-patient relationship facilitates the tailoring of therapeutic interventions to best suit individual patients’ needs [4]. Interpersonal skills honed over time through relational interactions with patients cannot be directly replaced by technology. Future technologies, including artificial intelligence, can only interpret what is explicitly documented as text or images in the electronic health record (EHR), which could lead to potentially missing valuable information generated within the physician-patient relationship, such as the patient’s values, preferences, trust, and rapport [5,6]. Moreover, a doctor’s personality and nonreflective actions, such as active listening or disregarding patient preferences, can have a significant impact not only on curing the patient’s illness but also on maintaining or exacerbating it [4]. Continuity of care with the same physician has been shown to reduce hospital admissions and mortality rates [7]. The rapid integration of technology introduces complex dimensions to an already intricate relationship, necessitating an exploration of technology’s impact on the physician-patient dynamic.

Digital technology offers potential for both improvements and risk, as it can efficiently reach a larger number of patients with less effort. At best, this technology-mediated efficiency may offer improved accessibility, real-time monitoring, and personalized treatments. It can also bridge geographical divides, connecting remote patients to specialists. At worst, it may be harmful on a scale far beyond what a single physician could achieve in a lifetime, potentially introducing systematic biases on a massive, automatic scale.

A potential problem with research on digital health technologies is the risk of bias, for example, when research funding comes from for-profit organizations and technology providers. As there may also be a dearth of independent research being published in this domain, we risk developing a skewed view of the evidence [8]. Additionally, while technological advancements surge ahead, ethical and regulatory frameworks have struggled to keep pace with these developments [9], making the need for new knowledge even more pressing.

Undeniably, digital technologies are here to stay and are already reshaping medical practice and the physician-patient relationship. Yet, more research is necessary to truly understand the nuanced implications of this shift. While technology promises efficiency and precision, the physician-patient relationship is rooted in intersubjective trust, empathy, and a deep understanding that transcends quantifiable data. As we further integrate technology into health care, it is therefore essential to explore its impact on this foundational relationship. Does it have the potential to augment the bond, creating new avenues for connection and understanding, or does it carry a more detrimental potential to create distance, becoming a screen that separates rather than connects human beings? By delving deeper into these questions, we can ensure that, as we advance technically, we do not lose sight of the human touch that remains at the heart of healing.

### Objectives

This review explores the existing knowledge gap concerning the impact of digital health on the physician-patient dynamic. Additionally, we analyze various theoretical frameworks and concepts used in empirical studies concerning this relationship and contrast the findings of these studies with the results of research grounded in relational ethics frameworks [10]. This research focus has enabled us to identify the consequences of using digital health technologies (such as digital messaging, telemedicine, health-related websites, and EHRs) in physician-patient relationships.

## Methods

### Overview

Due to the rapid advancements in digital health care, there is a noticeable knowledge gap in the existing literature. A scoping review was deemed a suitable review approach, as it is well suited for providing an updated understanding of the current state of knowledge [11]. This scoping review was conducted according to the guidelines of the PRISMA-ScR (Preferred Reporting Items for Systematic Reviews and Meta-Analyses extension for Scoping Reviews) [12]. The study protocol was preregistered with the Center for Open Science [13].

### Eligibility Criteria

The combined emergence of smartphones and 4G networks from around 2010 has played a crucial role in driving the development and use of digital health services [14]. This convergence has provided individuals with convenient access to health care services through mobile health (mHealth) apps, remote monitoring, and telemedicine, while also enabling health care providers to leverage real-time health data for personalized and data-driven care approaches. Only original peer-reviewed research published in English-language journals from January 1, 2010, to December 31, 2021, was included in this review. The decision to conclude the literature review on December 31, 2021, was driven by the aim to provide a comprehensive and current overview of the field up to our initial submission at the beginning of 2023. We carefully evaluated the scope and depth of available literature within this timeframe, determining that extending the search would likely not alter our core findings.

Research papers on patients and general practitioners or physicians involving health technology were included. Studies that did not specifically mention the physician-patient relationship were excluded.

### Information Sources

Studies for inclusion were identified through searches in the following databases: MEDLINE/PubMed, Embase, IEEE Xplore, and Cochrane. Since our review specifically targeted empirical research on the physician-patient relationship, we primarily focused on searching medical databases. However, we also included IEEE Xplore to ensure that we did not miss any significant research in the fields of computer science and electrical engineering. To ensure trustworthy findings and direct insights, our search prioritized original papers and solely focused on high-quality research papers published in peer-reviewed medical journals. Reviews, opinion pieces, and other nonempirical texts were not included. Duplicates were removed.

### Search

In collaboration with a librarian, we devised a comprehensive search string to explore the application of digital health technology within the physician-patient relationship. To ensure clarity in our scope, we used the mnemonic strategy PCC (population, concept, and context) to determine which papers should be included. Specifically, the population consisted of physicians, patients, or both; the concept involved digital health technologies used in medical care; and the context revolved around their implementation within the physician-patient relationship. The search string encompassed relevant terms, such as “digital health,” “mHealth,” “eHealth,” “telemedicine,” or “telehealth,” combined with the key phrases “physician-patient relations” or “relational ethics.” Detailed explanations of these terms are provided in Table 1. The search string used was (“digital health” OR “mHealth” OR “eHealth” OR “telemedicine” OR “telehealth”) AND (“physician-patient relations” OR “relational ethics”).

Our particular focus was on “relational ethics” due to its potential to inform a more ethical analysis of the physician-patient relationship, thus addressing the intricacies and challenges unique to this context.

We searched the 4 databases applying the following limitations: “scholarly (peer-reviewed) journals,” “date of publication from January 1, 2010 to December 31, 2021,” and “English language.” The search resulted in the identification of 429 journal papers. Of these papers, 427 were discovered in MEDLINE/PubMed, while 1 paper was identified as a duplicate appearing in both the Cochrane and MEDLINE/PubMed libraries. Additionally, 1 paper obtained from Embase was identified as a scoping review and was therefore excluded from our screening analysis. It is worth noting that no papers were found in IEEE Xplore during our search process. In short, all 427 papers selected for screening were found in the MEDLINE/PubMed database.

**Table 1:** Search terms.

Search terms	Explanation
digital health	Refers to technology related to health care services and solutions
mHealth^a^	Short for “mobile health,” involves the use of mobile devices
eHealth	Refers to “electronic health,” including digital health services
telemedicine	Involves remote diagnosis and treatment using technology
telehealth	Broad term covering health care services through telecommunication
Physician-patient relations	Refers to the interactions and dynamics between doctors and patients
relational ethics	Refers to an ethical framework focused on understanding and navigating the intricacies of interpersonal dynamics in the doctor-patient relationship

^a^mHealth: mobile health.

### Selection of Sources of Evidence

The screening process was performed by 2 researchers. It involved reviewing the abstracts initially found for inclusion, and in cases where abstracts lacked sufficient information, the entire research paper was examined. In instances where 2 researchers could not reach a consensus on whether to include a research paper, a third researcher was consulted as an arbiter to resolve any disagreements. This process led to the inclusion of 73 research papers. Subsequently, all 73 included papers underwent a detailed and thorough examination by 2 researchers. In the event of any disagreement during this phase, a third researcher was consulted to ensure accuracy. Studies that did not specifically mention the physician-patient relationship (n=63) were excluded, resulting in 10 studies to be reviewed.

### Data Charting Process

A standardized data charting form was created for the data extraction process, and data charting was performed by 2 reviewers.

### Data Items

Our objective was to collect a range of data items relevant to the research. These included the year of the study, the country where it was conducted, the study type (qualitative or quantitative), the theoretical or ethical framework and concepts used, the type of technology studied, the research objectives, and the participants involved (patients, physicians, and other health care personnel [HCP]; Table 2). Additionally, we extracted text samples that described the impact of digital health technologies on the physician-patient relationship. Our approach involved presenting the main findings in a textual manner.

**Table 2:** Overview of the data from the included studies.

Authors	Year	Journal	Country	Study type	Type of technology	Aims	Participants
Audrain-Pontevia and Menvielle [15]	2018	*Health Services Management Research*	Canada	Quantitative	Online health community	Examine how online health communities impact the physician-patient relationship	Patients
Balato et al [16]	2013	*British Journal of Dermatology*	Italy	Quantitative	Mobile phone messages	Evaluate the use of telemedicine in improving treatment adherence, patient outcomes, and the physician-patient relationship	Patients and physicians
Grünloh et al [17]	2018	*Journal of Medical Internet Research*	Sweden	Qualitative	Web-based patient portal	Investigate how physicians view the idea of patient participation	Physicians
Győrffy et al [18]	2020	*PLOS ONE*	Hungary	Qualitative	Social media	Explore physicians’ knowledge and attitudes toward digital health technologies and the transformation of the doctor-patient relationship	Physicians
Jiang [19]	2019	*Health Communication*	China	Quantitative	Digital messaging	Examine how the quality of face-to-face communication with providers is associated with their subsequent internet use for patient-provider communication	Patients
Kludacz-Alessandri et al [20]	2021	*PLOS ONE*	Poland	Qualitative	Teleconsultation (phone)	Study patients’ satisfaction with teleconsultation in primary care and the impact of teleconsultations on GP^a^-patient communication	Patients and physicians
Macdonald et al [21]	2018	*Journal of Medical Internet Research*	Canada	Qualitative	Digital messaging and EHR^b^	Examine HCP’s^c^ perspectives on how eHealth affects their relationships with patients, as well as its ethical ramifications	Patients, physicians, and other HCPs
Tasneem et al [22]	2019	*American Journal of Hospice and Palliative Medicine*	United States	Qualitative	Video consultation	Investigate the need for web-based videoconferences for oncology patients	Patients and physicians
Townsend et al [23]	2015	*Journal of Medical Internet Research*	Canada	Qualitative	Health-related websites	Focus on patients’ and HCP’s use of health-related internet information and how it influences the patient-HCP relationship	Patients, physicians, and other HCPs
Yan et al [24]	2020	*International Journal of Environmental Research and Public Health*	China	Qualitative	Digital messaging	Understand the underlying reasons for poor doctor-patient relationships	Patients and physicians

^a^GP: general practitioner.

^b^EHR: electronic health record.

^c^HCP: health care personnel.

### Synthesis of Results

To synthesize the results, we first created a summary based on the extracted data items to capture the key findings and statements from the included research papers concerning the physician-patient relationship. This summary was then compared with each research paper in its entirety to ensure accuracy.

Next, we conducted a narrative synthesis of the findings from the included studies, incorporating both the extracted text samples and the summaries of the papers. This approach allowed us to provide a comprehensive overview of the results from both quantitative and qualitative studies, enabling a comprehensive summary of the physician-patient relationship in the context of digital health technologies (Table 3). In addition, we assessed whether the papers applied any ethical frameworks or simple concepts and analyzed how their use contributed to the research (Table 4).

**Table 3:** Summary of evidence.

Authors	Summary: What does the paper state about the physician-patient relationship?
Audrain-Pontevia and Menvielle [15]	The paper discusses the impact of online health communities on the patient-physician relationship. The authors explore how online health communities, which provide users with computer-mediated social support and empowerment, impact this relationship. The authors acknowledge that, traditionally, doctors were the main source of medical information and therefore benefited from authority and power over their patients. However, with the advent of online health communities, patients now have access to social support, resources, and aid, which can make them feel more empowered and influence their relationships with their physicians. The authors propose that online health communities offer patients the opportunity to gain the power to handle their illnesses and their health, presumably leading to increased participation during the consultation and improving their commitment to their relationship with their physician.
Balato et al [16]	The study found that patient-physician communication improved in a group receiving SMS text message interventions, whereas it remained unchanged in the control group. This suggests that the use of digital interventions, such as SMS text messages, could potentially enhance the patient-physician relationship.
Grünloh et al [17]	The models of the doctor-patient relationship presented in the paper describe patients as being static and unchanged, but the authors note that patients with chronic conditions often encounter new situations and need to engage in a sensemaking and learning process. The paper suggests that the use of patient-accessible EHRs^a^ can contribute to the development of the doctor-patient relationship by allowing patients to play an active role. This increased patient participation makes it more difficult for physicians to maintain a strategy that potentially excludes patients. The authors state that eHealth does not have to be a “power struggle” in the doctor-patient relationship but can potentially help both partners improve their relationship collectively and grow individually. The authors mention the importance of patient participation for patient safety.
Győrffy et al [18]	The role of the doctor is in transition, with doctors expected to perform more complex tasks including health information technology and aiding in the digital orientation of patients. They see themselves transforming into mediators based on efficient communication with their patients. Digitally engaged physicians consider themselves guides, undertaking a guardian and information managing function in the description, collection, and sharing of credible content in the online space. For a successful leap from hierarchical patterns to the 21st-century doctor-patient relationship, the future generation of physicians should be trained differently and prepared for all the above-described changes. Medical school curricula should emphasize health and prevention rather than only diseases and pathology via the newest digital technological solutions. Medical students need to prepare for predictive and proactive working environments, including their new role as a guide or mediator for digitally empowered patients, in contrast with the paternalistic physicians of previous generations.
Jiang [19]	The results of the study emphasize the important roles of patient-centered communication and the physician-patient relationship in the eHealth and mHealth^b^ movement, particularly in the Chinese health care system. The interplay of physician-patient communication in face-to-face environments and relationship factors (eg, patient trust and patient satisfaction) could exert significant effects in promoting eHealth adoption. To encourage patients to adopt eHealth technologies, health care providers should first build a patient-centered environment (eg, responding to patients’ informational and emotional needs and engaging patients in medical decision-making).
Kludacz-Alessandri et al [20]	The study concerns patients’ satisfaction with teleconsultation in primary care and the impact of teleconsultations on GP^c^-patient communication during the COVID-19 pandemic in Poland. The paper suggests that the quality of GP-patient communication is an essential factor that can improve the results of treatment and patient satisfaction. Only 55% (n=99) of the patients found teleconsultation to be as good as in-person visits with their physician.
Macdonald et al [21]	The study discusses the concept of a “two-way conversation” that is evolving in the health care provider-patient dynamic. This shift toward more collaborative interactions with patients is, in part, facilitated by eHealth technologies. The authors examine the impact of eHealth on the current state of collaborative consultation, highlighting how it aids in having, using, and supporting conversations with patients. Some health care professionals in the study embraced the idea of patients as “partners,” as they see a partner as someone who helps in improving an outcome by educating themselves and conscientiously monitoring their condition and behavior. One of the health care professionals stated that patients who are engaged through eHealth and informed about their condition are more useful clinically. The paper also mentions a pedagogical approach to interacting with the eHealth users among patients.
Tasneem et al [22]	The study assessed the needs of patients receiving palliative care and their perception of how telemedicine video visits might influence their care. Despite concerns about truncated physical examinations and prescription limits, the majority of patients favored having the opportunity for telemedicine video visits. They felt that the physician-patient relationship would not diminish and had few cost concerns. They believed that a video alternative to an in-person visit might increase access, save time, and increase comfort and safety by avoiding a trip to the hospital.
Townsend et al [23]	In this study, patients reported using personal websites, blogs, chat rooms, and online links to medical test results as part of their eHealth resources. This suggests that patients are actively engaging with digital resources in managing their health, which can have implications for the physician-patient relationship. The paper discusses how the rapid explosion in online digital health resources is seen as transformational, accelerating the shift from traditionally passive patients to patients as partners. This is altering the current patient-health care professional relationship. The proliferation of eHealth strategies is accelerating a shift in health care from a traditional and paternalistic delivery model to a more mutual patient-health care professional relationship in which informed patients are actively involved in their care and treatment decisions. The authors mention that eHealth resources provide patients with extensive and up-to-date information, access to medical research, connections to people with similar conditions, immediacy, and convenience in patient-health care professional communications. These factors can significantly impact the physician-patient relationship.
Yan et al [24]	The paper investigates the underlying reasons for poor doctor-patient relationships in mobile consultation from the perspective of computer-mediated communication. This suggests that the physician-patient relationship may be influenced by the mode of communication, particularly in a digital context. The paper emphasizes the emerging use of mobile medical consultation in China, which has propelled the establishment of doctor-patient relationships in the mobile context. This again underscores the impact of digital technologies on the physician-patient relationship. The authors also mention different models or concepts used to assess the doctor-patient relationship, suggesting that the nature of this relationship can be complex and multifaceted.

^a^EHR: electronic health record.

^b^mHealth: mobile health.

^c^GP: general practitioner.

**Table 4:** Theoretical frameworks and concepts used in the studies.

Authors	Framework or concepts	Relevance of using specific theoretical frameworks and concepts
Audrain-Pontevia and Menvielle [15]	Patient empowerment	Evaluates the impact of online health communities on the physician-patient relationship.
Balato et al [16]	None mentioned	N/A^a^
Grünloh et al [17]	Shared decision-making, patient-centered care, and paternalism	Assesses the impact of a web-based patient portal on patient participation.
Győrffy et al [18]	Patient empowerment and shared decision-making	Studies the attitudes of digitally engaged physicians toward transforming the physician-patient relationship.
Jiang [19]	Patient-centered care, mutual trust, and patient satisfaction	Examines the relationship between face-to-face and online patient-provider communication.
Kludacz-Alessandri et al [20]	Mutual trust	Investigates the satisfaction of patients with teleconsultations.
Macdonald et al [21]	Relational ethics	Relational ethics addresses the ethical content and decisions implicit in everyday relationships and conversations.
Tasneem et al [22]	None mentioned	N/A
Townsend et al [23]	Relational ethics	Core elements of relational ethics are applicable to everyday experiences, practice, and interactions. Applying relational ethics helps with focusing on what is valued in interactions and relationships and what is at risk rather than specific aspects of eHealth such as the nature of self-monitoring devices.
Yan et al [24]	None mentioned	N/A

^a^N/A: not applicable.

## Results

### Selection of Sources of Evidence

**Figure 1:**
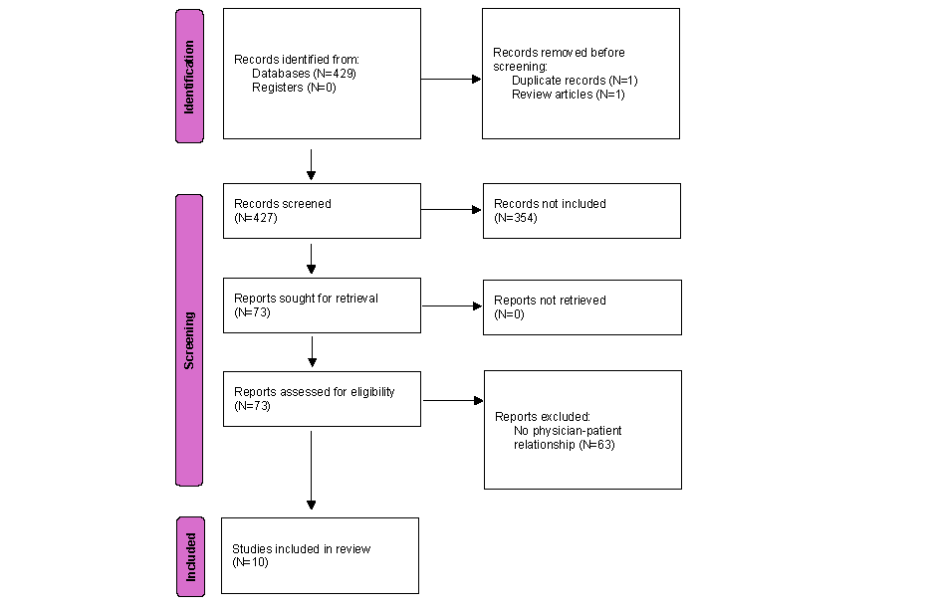
PRISMA (Preferred Reporting Items for Systematic Reviews and Meta-Analysis) diagram: papers included in the review.

Out of the 427 studies screened, 73 studies were sought out for retrieval, and after excluding 63 studies that did not focus on the physician-patient relationship, 10 studies were included in this review. Figure 1 illustrates the search process.

### Characteristics of Sources of Evidence

The research papers included in this study [15-24] were conducted in various countries, with 3 in Canada, 2 in China, 1 in Italy, 1 in Sweden, 1 in Hungary, 1 in Poland, and 1 in the United States. These papers were published between 2013 and 2021, with half of them published in 2019 or more recently. The majority of the papers (n=7) were qualitative studies, with the remaining (n=3) papers being quantitative studies. Only 2 research papers, authored by Macdonald et al [21] and Townsend et al [23], referred to a specific ethical framework. Both used relational ethics. A total of 6 other papers adopted different theoretical frameworks and concepts, while the remaining 2 did not use any theoretical frameworks at all.

Regarding the technologies investigated, 3 papers focused on online communities, 3 papers focused on messaging, 2 papers focused on telephone or video consultation, and 2 papers focused on patient portals or EHR. The majority of the papers (n=6) included a mix of patients and physicians or other health care professionals, while 2 papers focused solely on patients and 2 others exclusively on physicians.

For further details about the characteristics of the included research papers, please refer to Table 2.

### The Impact of Digital Health Technologies on the Physician-Patient Relationship

Kludacz-Alessandri et al [20] noted that, while teleconsultations offer convenience and safety, especially in situations like the COVID-19 pandemic, they may not always provide the same level of patient satisfaction as in-person visits. According to the authors, this could be due to several reasons such as the inability to read nonverbal cues, technical difficulties, or the impersonal nature of digital communication. The authors suggested that, while digital technologies offer new avenues for communication, they may not fully mirror the richness and depth of face-to-face interactions.

Yan et al [24] discussed the use of mobile medical consultation in China and noted the potential challenges in establishing effective physician-patient relationships in a digital context. According to the authors, 1 key challenge could be the varying degrees of technology literacy among patients. Not all patients may be comfortable using digital platforms for health care, and some may lack access to the necessary technology. This study indicates that this could lead to inequities in access to health care services and affect the quality of the physician-patient relationship.

When patients become more empowered through access to digital health resources and online communities, changes could occur in the power dynamics of the physician-patient relationship. Some physicians may struggle to adapt to these changes, particularly if they are trained in a more traditional, paternalistic model of health care. This was suggested in the study by Audrain-Pontevia and Menvielle [15].

The advent of online health communities and access to a wealth of health-related information on the internet has empowered patients to take an active role in managing their health. As discussed by Audrain-Pontevia and Menvielle [15], this empowerment can lead to increased participation during consultations and improve commitment to the relationship with the physician.

Digital technologies, such as SMS text messaging, teleconsultations, and mHealth apps, can enhance communication between patients and physicians, making it more frequent, timely, and convenient. As pointed out by Balato et al [16], such digital interventions can potentially enhance the patient-physician relationship.

The use of eHealth and mHealth technologies facilitates a more patient-centered approach to care. As Jiang [19] noted, health care providers who build a patient-centered environment—responding to patients’ informational and emotional needs and engaging them in medical decision-making—can promote eHealth adoption and improve health care outcomes.

Digital health technologies can improve the efficiency of health care delivery and make health services more accessible. For example, Tasneem et al [22] found that patients receiving palliative care favored the opportunity for video consultations, as they could save time while increasing access, comfort, and safety by avoiding a trip to the hospital.

eHealth resources provide patients with extensive and up-to-date information, access to medical research, and connections to people with similar conditions. Townsend et al [23] mentioned that these factors can significantly impact the physician-patient relationship, transforming patients from passive recipients of care into active partners in their health care journey.

The availability of health information online and through digital technologies enables patients to be more informed about their health conditions and treatment options. This can lead to better-shared decision-making between patients and physicians [17,18,21].

### The Application of Theoretical Frameworks and Concepts

In the study by Grünloh et al [17], the use of 2 theoretical frameworks (shared decision-making and patient-centered care) and 1 concept of the physician-patient relationship (paternalism) helped highlight the roles of the medical professional and the patient, as well as the ways in which patients can contribute to the relationship.

The concept of patient empowerment (used in the study by Audrain-Pontevia and Menvielle [15]) helped with understanding how online health communities influence the patient-physician relationship, but it did not capture the complexities of the power dynamics and relational aspects inherent in these interactions.

Győrffy et al [18] focused on the attitudes of digitally engaged physicians toward transforming the physician-patient relationship. The researchers used the theoretical frameworks of patient empowerment and physician-patient collaboration to assess current and ideal physician-patient relationships. Through this, they recognized that the digital age requires physicians to transition from a role of authority to one of guidance.

Jiang [19] examined the relationship between face-to-face and online patient-provider communication through the concepts of patient-centered care, trust, and satisfaction.

Kludacz-Alessandri et al [20] investigated patients’ satisfaction with teleconsultations by considering the concepts of respect and dialogue. These concepts are indeed valuable in understanding the impacts of teleconsultations on the physician-patient relationship.

The 3 studies that did not apply any theoretical frameworks had either superficial findings regarding the physician-patient relationship or it was not clear how they reached their conclusions. For example, Balato et al [16] simply quantified the physician-patient relationship through a 10-point scale questionnaire. Tasneem et al [22] asked patients only about how the technology would affect their relationship with their physician in 2 out of 15 questions. Finally, Yan et al [24] seemed to apply the concepts of mutual respect and patient satisfaction in a deep and meaningful way, but we have no way of knowing since they do not specifically state what concepts they used.

### The Application of Relational Ethics

In contrast with the above-applied frameworks, the use of a relational ethics framework can help reveal the subtle and complex impacts of digital health technologies on the physician-patient relationship. For example, in the study by Macdonald et al [21], a relational ethics lens was used to examine how eHealth technology contributes to changes in relations between HCPs and patients, evolving toward more collaborative care. By focusing on every day relationships and conversations, the study was able to understand how these technologies incorporated the relational ethics of patient-centered care into practice.

Relational ethics can help address the power dynamics and vulnerabilities that come into play in the physician-patient relationship, especially with the use of digital technologies. In the study by Townsend et al [23], relational ethics was used to understand how technology impacts relational shifts in ethical patient-HCP relationships. They found that technology use could lead to a transition from a traditional hierarchical relationship to a more reciprocal relationship, which could reveal mutual vulnerabilities.

## Discussion

### Principal Results

The studies included in this review were diverse, with research conducted across various countries and contexts and exploring a range of technologies, such as online communities, messaging, teleconsultations, and patient portals or EHRs. In terms of user groups, they examined the experiences of patients, physicians, and other HCPs, offering a comprehensive view of the phenomenon from different user perspectives.

Through the review, we identified several consequences of using digital health technologies in health care. The convenience and accessibility offered by these technologies have the potential to transform the health care landscape by enhancing communication, improving efficiency, and empowering patients. However, they also provide challenges and complexities such as increasing inequities in access to health care services due to variations in technology literacy among patients and decreases in patient satisfaction due to limitations in nonverbal communication and the impersonal nature of digital interactions.

The review further highlighted the shift in power dynamics in the physician-patient relationship from a traditional hierarchical model toward a more reciprocal and collaborative model. This shift is facilitated by the empowering potential of digital health technologies and online health communities but may also present challenges for physicians trained in a more paternalistic model of approach to medicine.

In addition to mapping key findings, we analyzed the theoretical frameworks used in the studies to contrast the use of relational ethics with the application of other theoretical frameworks. Of the 10 studies in this review, 2 did not apply any theoretical frameworks to their research. Others used frameworks or concepts such as patient-centered care, patient empowerment, or shared decision-making. Only 2 studies used relational ethics as their framework.

Relational ethics, by its very nature, emphasizes the value of intersubjective qualities such as empathy, trust, respect, and mutual responsibility. These qualities are critical to the practice of effective and compassionate health care, and their importance is highlighted in the context of digital health technologies interjected between the physician and the patient. While theoretical frameworks and concepts, such as patient-centered care and patient empowerment, provided valuable insights into the impacts of digital health technologies, a relational ethics perspective could provide a more comprehensive understanding. For instance, it could explore how trust is built and maintained in online versus face-to-face interactions, how the power dynamics between physicians and patients might shift in an online context, and how empathy and understanding are conveyed through digital mediums. A relational ethics framework could further enrich this understanding by examining how respect and dialogue contribute to a sense of mutual trust and understanding, how they shape the power dynamics in a teleconsultation, and how they can foster a sense of connection and empathy in a digital environment.

### Comparison to Prior Work

This review underscores the potential value of using a relational ethics framework in research on the physician-patient relationship in the complex real-life context of digital health technologies. While previous works have acknowledged the importance of relational aspects in health care delivery [3,5], few have explicitly used relational ethics as a framework to examine the nuanced ethical implications of digital health technologies on the physician-patient relationship.

### Limitations and Strengths of This Study

Because this review amassed studies from a variety of countries, we acknowledge that the specific cultural, societal, and health care contexts of these regions can have a significant effect on the physician-patient relationship and the use and adoption of digital health technologies. As such, our findings might not be applicable everywhere.

The review only included studies published in English, which could introduce language bias. If there were relevant studies published in other languages, they would have been left out, potentially narrowing the range of perspectives and findings we were able to consider.

While our review touched on a selection of digital health technologies—including online communities, messaging services, teleconsultations, and patient portals or EHRs—many more kinds of technology are being used in health care. As such, our findings are limited to the specific digital health technologies covered in this review.

Scoping reviews, like this one, are primarily intended to provide a broad overview of the existing literature rather than evaluate the strength of evidence or perform a meta-analysis. This means that it is difficult to draw definitive conclusions or provide firm recommendations based on our findings.

While our review encapsulates literature up to the end of 2021, reflecting the state of research at the time of our initial submission, it may not include the latest developments or studies published post-2021.

The quality of the studies included in a review can have a large effect on its findings. Since we did not conduct a critical appraisal of the quality of the studies we included, this could be seen as a limitation.

### Conclusions

Overall, this review offers a comprehensive overview of the current state of evidence regarding the impacts of digital health technologies on the physician-patient relationship. It underscores the potential of these technologies to transform health care delivery, while also highlighting the challenges and complexities they introduce. The review emphasizes the need for further research using a relevant ethics framework to provide a deeper understanding of the impact of digital health technologies on the physician-patient relationship. This will be particularly crucial as digital health technologies continue to expand, evolve, and become more integrated into health care delivery.

## Appendix

Multimedia Appendix 1 PRISMA-ScR (Preferred Reporting Items for Systematic Reviews and Meta-Analyses extension for Scoping Reviews) checklist.

## Abbreviations

EHR: electronic health record
HCP: health care personnel
mHealth: mobile health
PCC: population, concept, and context
PRISMA-ScR: Preferred Reporting Items for Systematic Reviews and Meta-Analyses extension for Scoping Review
